# Supplementary material for: Comprehensive multi-omics analysis reveals WEE1 as a synergistic lethal target with hyperthermia through CDK1 super-activation
Source: Nat Commun. 2024 Mar 7;15:2089. doi: 10.1038/s41467-024-46358-w (PMC10920785; doi:10.1038/s41467-024-46358-w)
Supplement: Supplementary file 1 — Supplementary Information [file 41467_2024_46358_MOESM1_ESM.pdf]

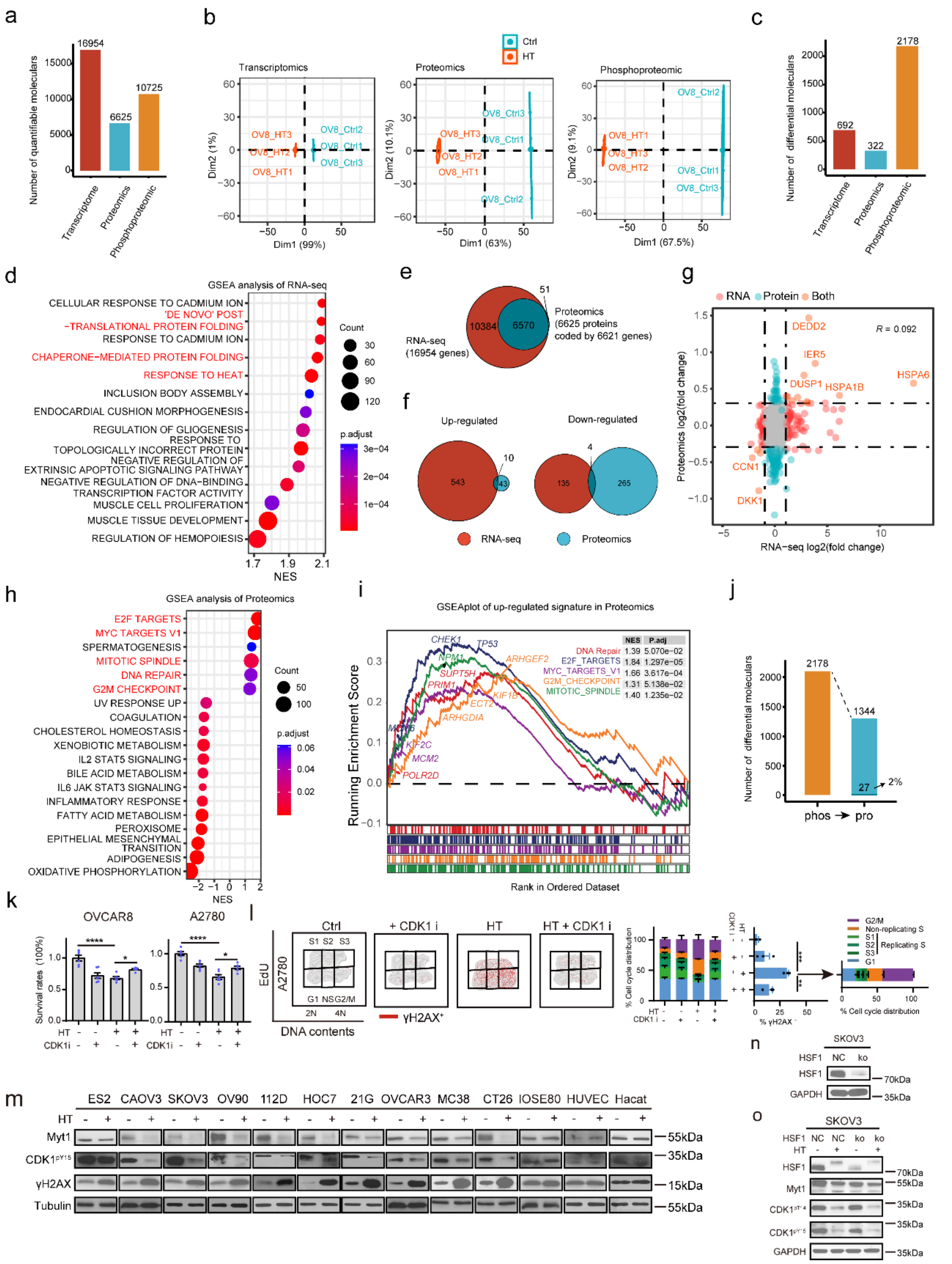


**Fig. S1, related to Fig. 1**

**a** Bar plot showing the number of genes quantified in transcriptomics, proteomics, and phosphoproteomics. **b** PCA plots showing the separation of transcriptomics, proteomics, and phosphoproteomics after hyperthermia (HT). **c** Differential expression

analysis of RNA, protein, and phosphorylation levels after HT. **d** GSEA analysis was executed to identify major changes in transcriptomics after HT. **e** Venn diagram showing the overlap of quantifiable proteins in proteomics and mRNA in transcriptomics. **f** Venn diagrams showing the overlap of molecules that were up-regulated (left) or down-regulated (right) after HT in transcriptomics and proteomics. **g** Scatter plot showing the overlap and correlation between protein and RNA expression changes. Significant changes at RNA (red), protein (blue), and both levels (orange, correlation coefficient R = 0.092) are shown respectively. **h** GSEA analysis was performed using proteomics. **i** GSEA plot showing the signaling pathways significantly perturbed after HT using proteomics. **j** Bar plot showing the number of proteins and differential proteins corresponding to the significantly regulated phosphosites upon HT (2% of differentially phosphorylated proteins exhibited change at the protein level). **k** Changes in cell viability were determined by CCK8 assay after exposure to HT in the presence of CDK1 inhibitor or not (n=6 per group). **l** γH2AX, EDU, and PI positive cells were analyzed by flow cytometry in A2780 cells after exposure to HT in the presence of CDK1 inhibitor or not (n=3 per group). **m.** Representative images validating phosphoproteomics by western blot in the indicated cells. **n** Representative images of western blot analysis depicting HSF1 expression in CRISPR-Cas9 mediated HSF1-knockout clones of SKOV3 cells. **o** Representative western blot images depict the expression levels of indicated proteins in Scramble-knockout (NC) or HSF1-knockout (HSF1-ko) SKOV3 cells after exposure to either 37 °C or 42 °C. Comparisons were performed by one-way ANOVA followed by Dunnett's multiple comparisons test in (**l**) and one-way ANOVA followed by Tukey's multiple comparisons test in (**k**). Data are presented as mean ± SEM. ****$p < 0.0001$, ***$p < 0.001$, **$p < 0.01$, *$p < 0.05$. All analytical data are derived from a minimum of three biologically independent experiments, with 'n' indicating the specific number of replicates. Source data are provided as a Source Data file.

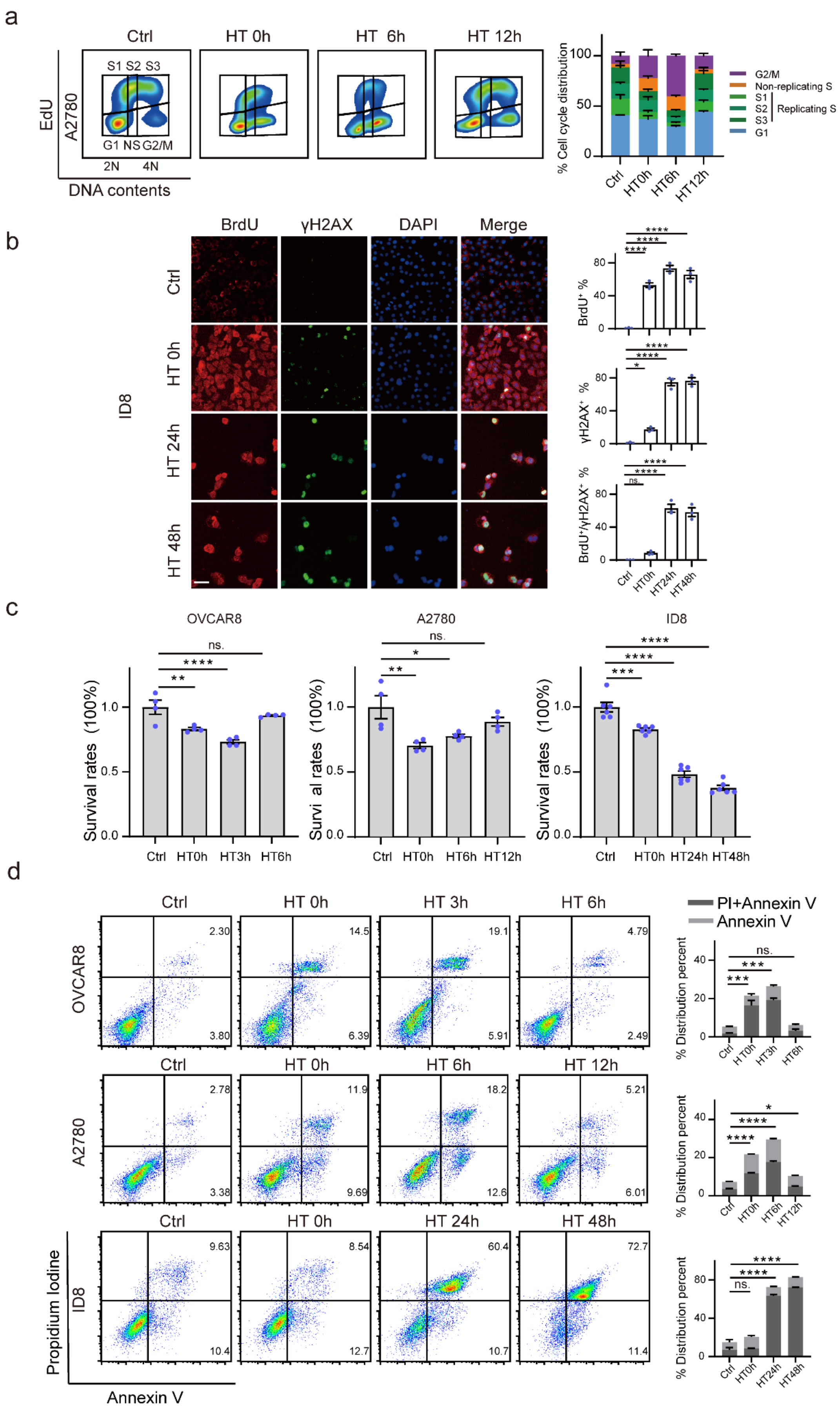
a
Ctrl
HT 0h
HT 6h
HT 12h
EdU
A2780
S1 S2 S3
G1 NS G2/M
2N 4N
DNA contents
% Cell cycle distribution
G2/M
Non-replicating S
S1
S2
S3
G1
Replicating S
b
BrdU
γH2AX
DAPI
Merge
ID8
HT 24h
HT 48h
BrdU⁺ %
γH2AX⁺ %
BrdU⁺/γH2AX⁺ %
c
OVCAR8
A2780
ID8
Survival rates (100%)
Ctrl HT0h HT3h HT6h
Ctrl HT0h HT6h HT12h
Ctrl HT0h HT24h HT48h
d
PI+Annexin V
Annexin V
OVCAR8
A2780
ID8
Propidium Iodine
Annexin V
% Distribution percent

**Fig. S2, related to Fig. 3**

**a** Cell cycle was analyzed by flow cytometry in A2780 cells treated as in Fig 3b (n=3 per group). **b** Representative images and quantification of BrdU and γH2AX positive ID8 cells treated as in Fig 3b (n=3 per group). Scale bar, 25 μm. **c** Cell viability was determined by CCK8 assay in cells treated as indicated (ID8: n=6 per group; OVCAR8 and A2780: n=4 per group). **d** Apoptosis was analyzed by flow cytometry in cells treated as indicated (n=3 per group). Comparisons were performed by one-way ANOVA followed by Dunnett's multiple comparisons test in (**b, c, d**) Data are presented as mean ± SEM. ****p < 0.0001, ***p < 0.001, **p < 0.01, *p < 0.05, ns., non-significant. All analytical data are derived from a minimum of three biologically independent experiments, with 'n' indicating the specific number of replicates. Source data are provided as a Source Data file.

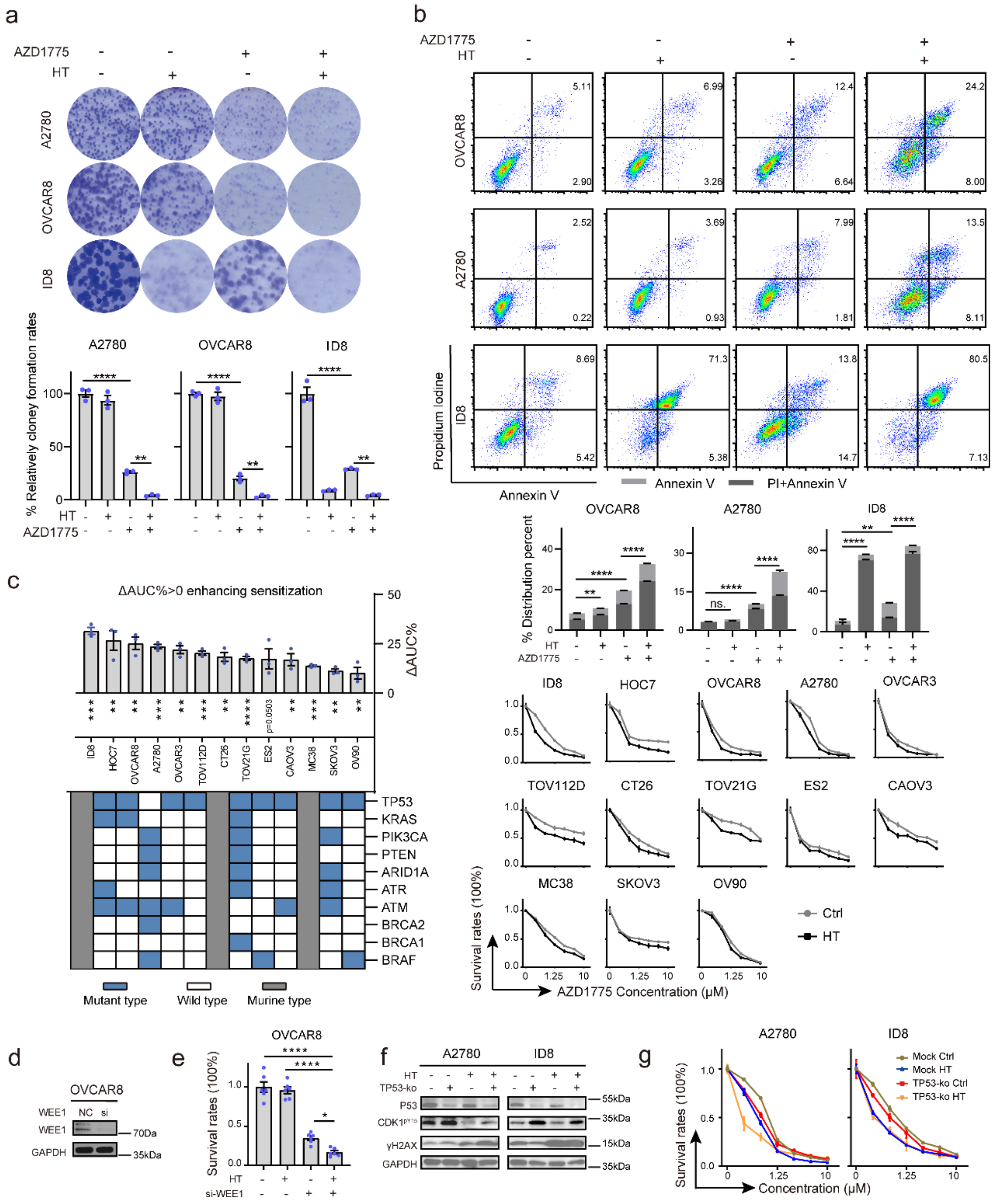
a
AZD1775
HT
A2780
OVCAR8
ID8
% Relatively cloney formation rates
b
Propidium Iodine
Annexin V
Annexin V
PI+Annexin V
% Distribution percent
c
ΔAUC%>0 enhancing sensitization
ΔAUC%
ID8
HOC7
OVCAR8
A2780
OVCAR3
TOV112D
CT26
TOV21G
ES2
CAOV3
MC38
SKOV3
OV90
p=0.0503
TP53
KRAS
PIK3CA
PTEN
ARID1A
ATR
ATM
BRCA2
BRCA1
BRAF
Mutant type
Wild type
Murine type
Survival rates (100%)
AZD1775 Concentration (μM)
Ctrl
HT
d
OVCAR8
WEE1
NC
si
GAPDH
70Da
35kDa
e
si-WEE1
f
TP53-ko
P53
γH2AX
55kDa
15kDa
g
Mock Ctrl
Mock HT
TP53-ko Ctrl
TP53-ko HT
Concentration (μM)

h

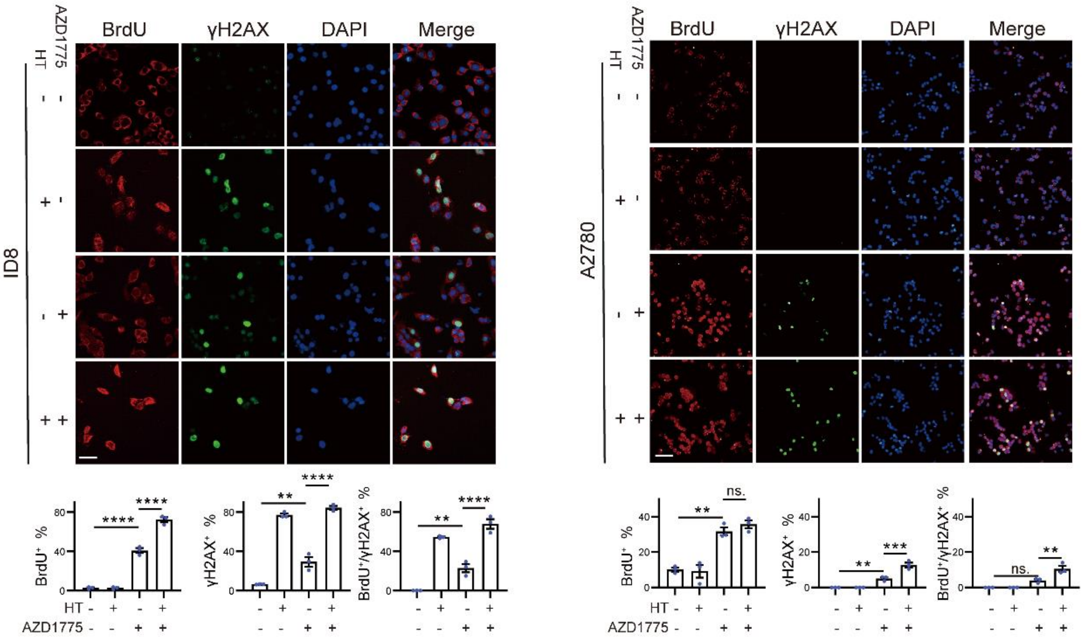


i

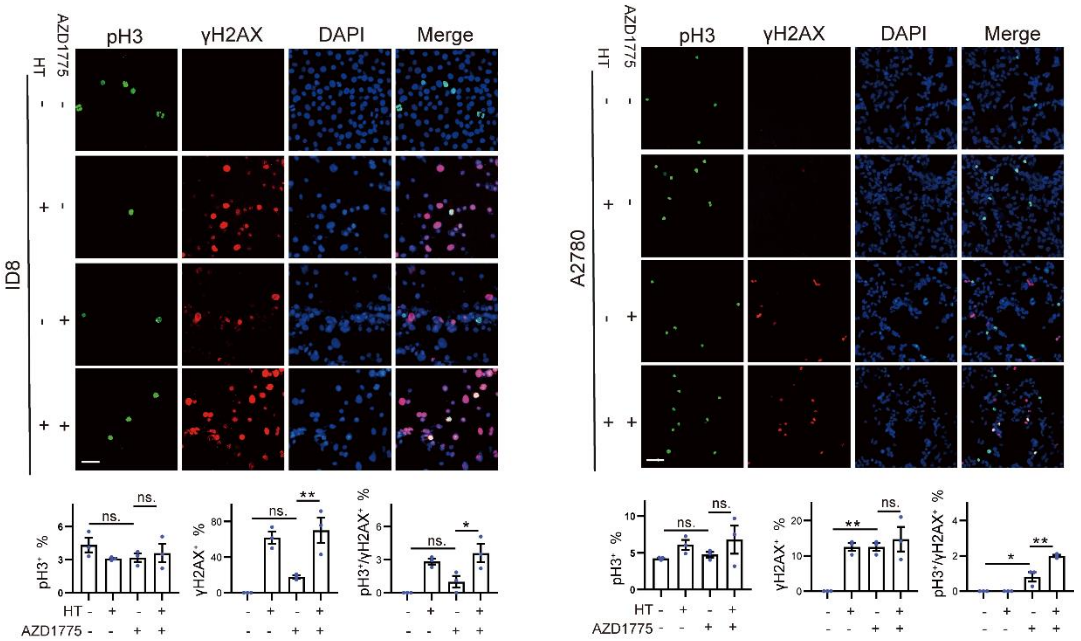


**Fig. S3**, **related to Fig. 4**

**a** Representative images of clonogenic assays in A2780, OVCAR8, and ID8 treated as in Fig 4e for 8 days (n=3 per group). **b** Flow cytometry results for apoptosis in OVCAR8, A2780, and ID8 cells, as treated in Fig 4e (n=3 per group). **c** ΔAUC% (left) and dose-response curves (right) of AZD1775 mono-therapy or HT-AZD1775 concurrent therapy in cancer cells with the indicated genetic background profile (n=3 per group). **d** Representative images of western blot analysis illustrating WEE1

expression in OVCAR8 cells transfected with scramble siRNA (NC) or WEE1 siRNA. **e** Cell viability was determined by CCK8 assay in OVCAR8 cells treated as indicated (n=6 per group). **f** Representative images of western blot analysis illustrating P53, CDK1-pY15, and γH2AX proteins in of A2780 and ID8 cells or CRISPR-Cas9 mediated TP53-knockout (TP53-ko) clones. **g** Dose-response curves for AZD1775 mono-therapy or HT-AZD1775 concurrent therapy in A2780/ID8 cells (Mock) or TP53-ko clones (n=3 per group).**h** Representative images and quantification of BrdU and γH2AX positive ID8 and A2780 cells treated as in b (n=3 per group). Scale bar, 25 μm. **i** Representative images and quantification of pH3 and γH2AX positive ID8 and A2780 cells treated as in b (n=3 per group). Scale bar, 25 μm. Comparisons were performed by unpaired two-tailed Student's t-test in (**c**), one-way ANOVA followed by Dunnett's multiple comparisons test in (**e**), and one-way ANOVA followed by Tukey's multiple comparisons test in (**a, b, h, i**). Data are presented as mean ± SEM. ****$p < 0.0001$, ***$p < 0.001$, **$p < 0.01$, *$p < 0.05$, ns., non-significant. All analytical data are derived from a minimum of three biologically independent experiments, with 'n' indicating the specific number of replicates. Source data are provided as a Source Data file.

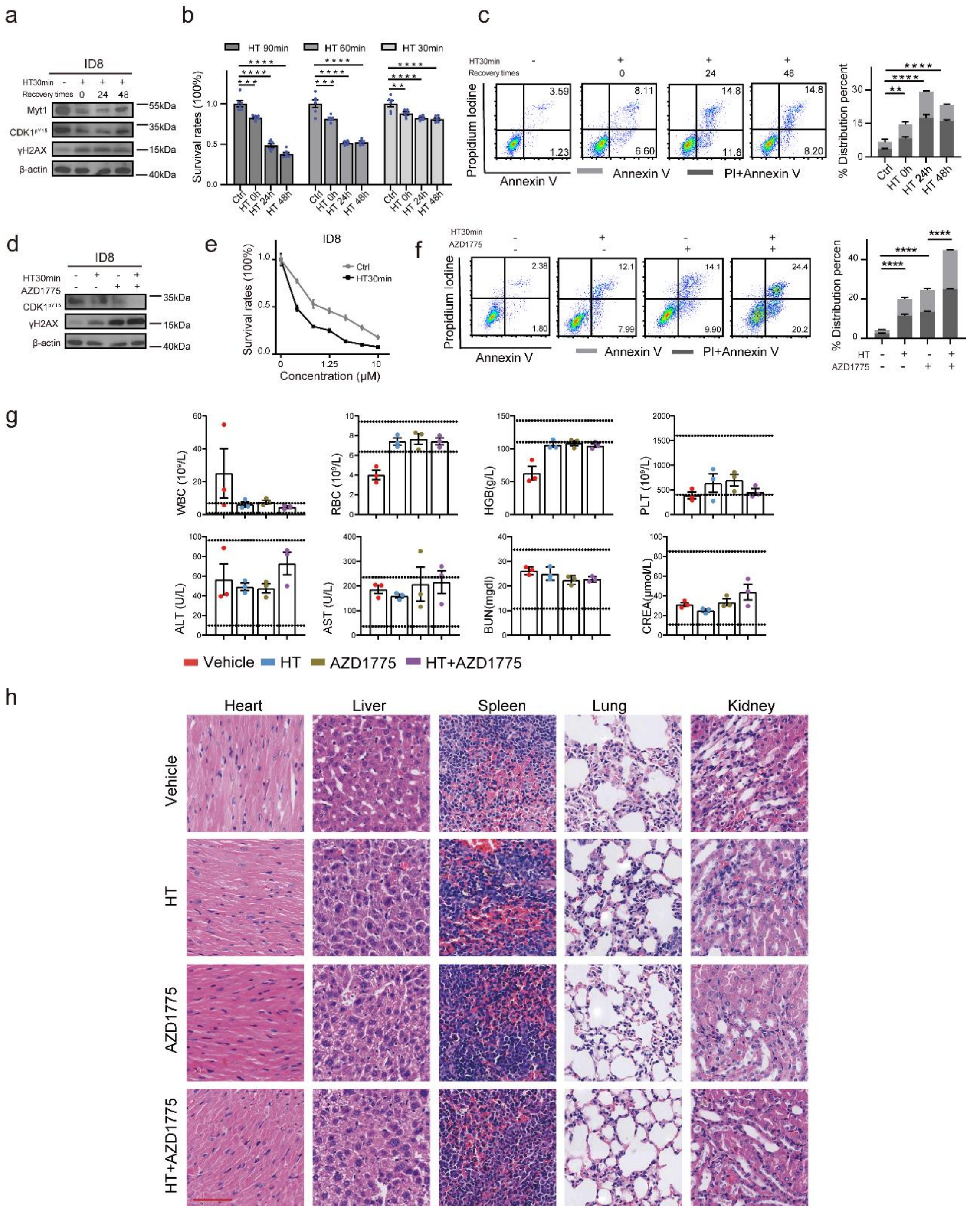


## Fig. S4, related to Fig. 8

**a** Representative images of western blot analysis depict the effects of hyperthermia (HT) incubation for 30 min on Myt1, CDK1-pY15, and γH2AX proteins in ID8 cells. **b** Cell viability was measured by CCK8 in ID8 cells treated as indicated (n=6 per group). **c** Apoptosis was analyzed by flow cytometry in ID8 cells treated as in a (n=3 per group). **d** Representative images of western blot with indicated proteins. ID8 cells were treated

with AZD1775 (800 nM) for 48 h in the presence or absence of HT for 30 min.**e** Cell viability was measured by CCK8 in cells treated with AZD1775 mono-therapy or HT (30min)-AZD1775 concurrent therapy (n=3 per group). **f** Apoptosis was analyzed by flow cytometry in cells treated as in d (n=3 per group). **g** A  A graphical representation displaying alanine aminotransferase (ALT), aspartate aminotransferase (AST), blood urea nitrogen (BUN), creatinine (CREA), white blood cell (WBC), red blood cell (RBC), hemoglobin (HGB), and platelet (PLT) levels in mice subjected to different treatments (n=3 per group). Dotted lines denote the reference range. **h** Toxicity assessment with pathological sections: histological examinations of HE-stained heart, liver, spleen, lung, and kidney. Scale bar, 50 μm. Comparisons were performed by one-way ANOVA followed by Dunnett's multiple comparisons test in (**b, c**) and one-way ANOVA followed by Tukey's multiple comparisons test in (**f**). Data are presented as mean ± SEM. ****p < 0.0001, ***p < 0.001, **p < 0.01. A  All analytical data are derived from a minimum of three biologically independent experiments, with 'n' indicating the specific number of replicates. Source data are provided as a Source Data file.

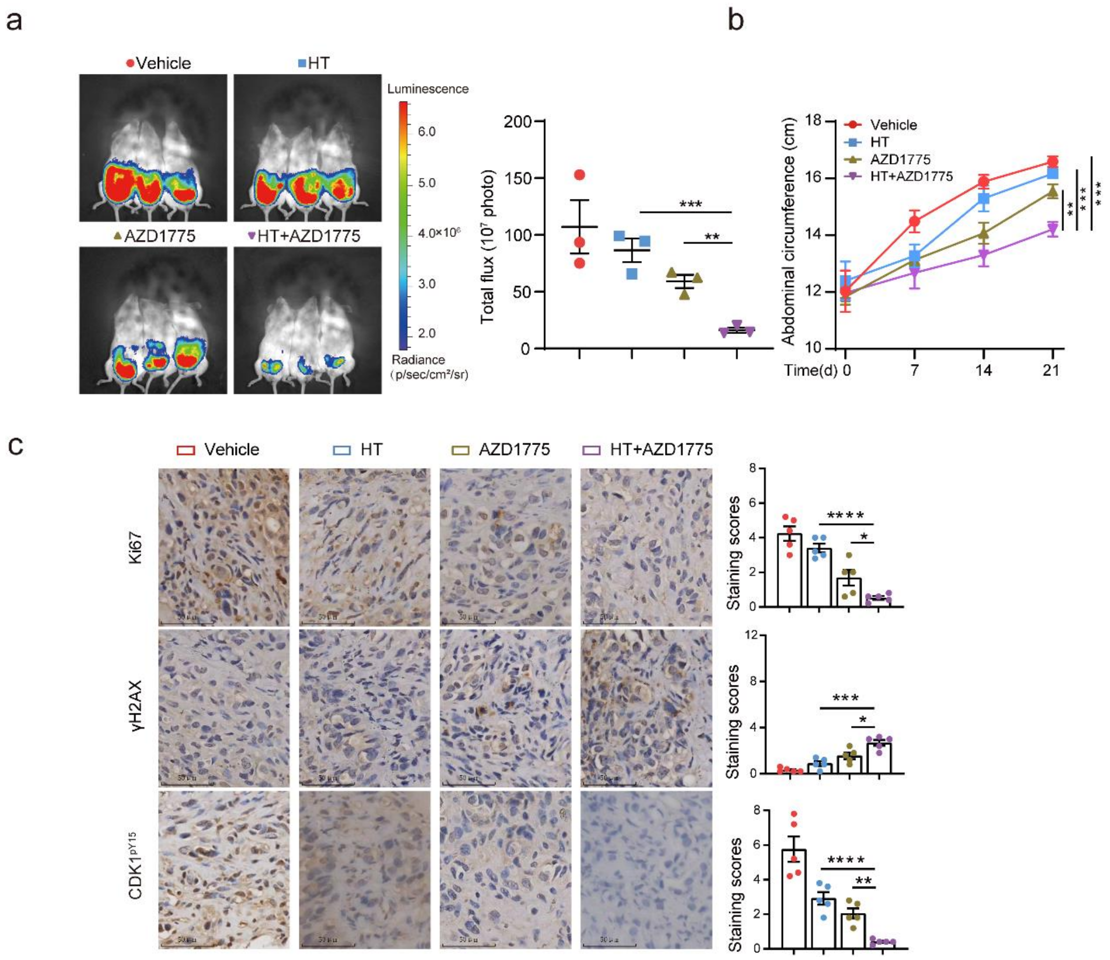


**Fig. S5, related to Fig. 8**

**a** Representative image (left) and quantification of bioluminescence (right) in NOD mice after treatment as in Fig 8b (n=3 mice per group). **b** Quantification and statistical analysis of the abdominal circumference of NOD mice, recorded every 7 days (n=3 per group). **c** Tissue sections were stained with Ki67, γH2AX, and CDK1-pY15 (n=5 samples from 3 mice per group). Scale bar, 50 μm. The quantification of IHC staining scores is shown in the right panel. Comparisons were performed by one-way ANOVA followed by Dunnett's multiple comparisons test in (**a, b, c**). Data are presented as mean ± SEM. ****p < 0.0001, ***p < 0.001, **p < 0.01, *p < 0.05. All analytical data are derived from a minimum of three biologically independent experiments, with 'n' indicating the specific number of replicates. Source data are provided as a Source Data file.

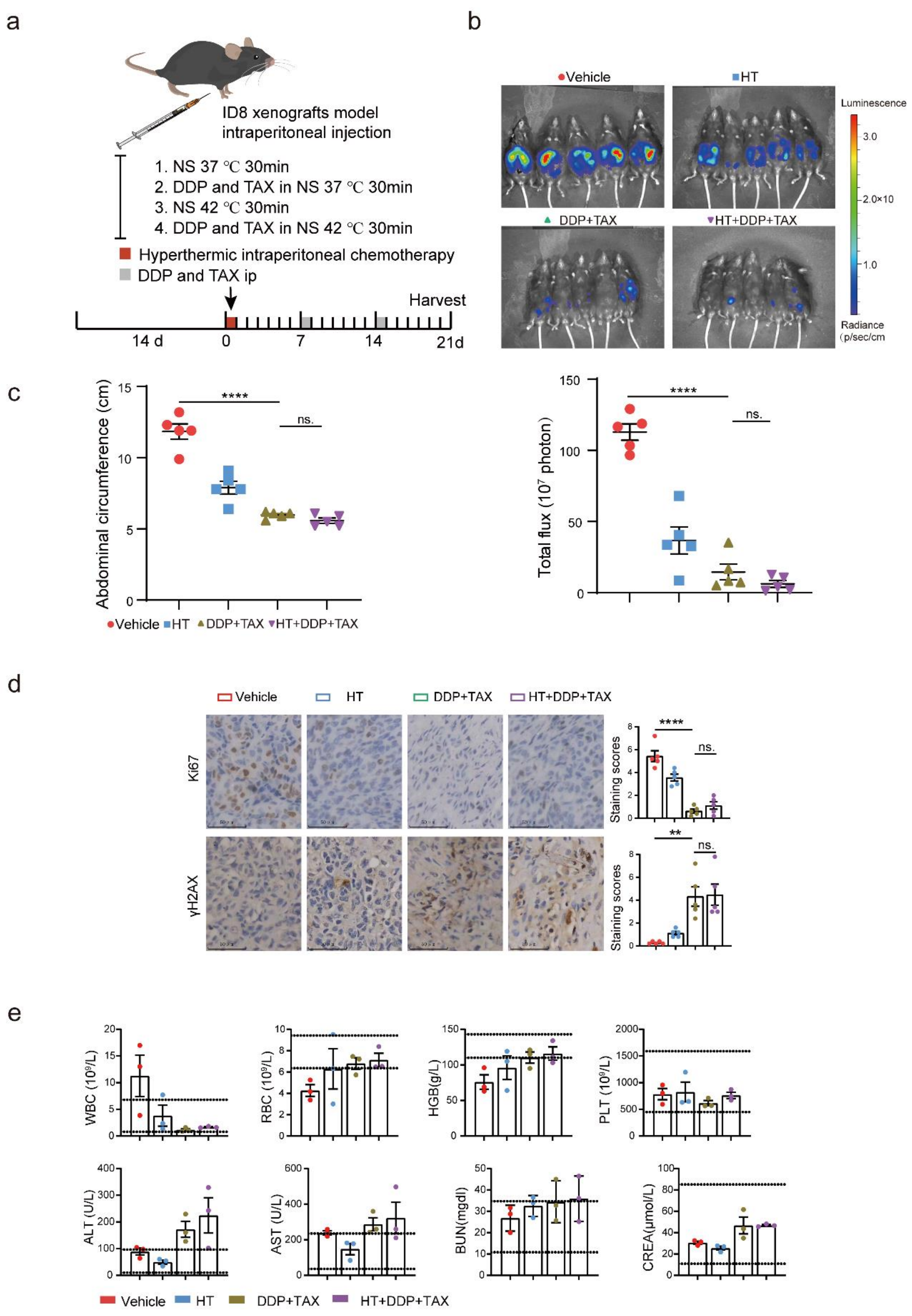


**Fig. S6, related to Fig. 8**

**a** Schema of the mouse HIPEC protocol. Third-party elements were modified from the Scidraw (https://scidraw.io/), licensed under a Creative Commons Attribution 4.0

Generic License (https://creativecommons.org/licenses/by/4.0/). **b** Representative images (above) and quantification of bioluminescence (below) in C57BL/6 mice (n=5 mice per group). **c** Quantification and statistical analysis of the abdominal circumference of mice (n=5 per group). **d** Tissue sections were stained with Ki67 and γH2AX (n=5 per group). Scale bar, 50 μm. The quantification of IHC staining scores is shown in the right panel. **e** A plot of ALT, AST, BUN, CREA, WBC, RBC, HGB, and PLT levels in mice following various treatments (n=3 per group). Dotted lines indicate the reference range. Comparisons were performed by one-way ANOVA followed by Dunnett's multiple comparisons test in (**b, c**) and one-way ANOVA followed by Tukey's multiple comparisons test in (**d**). Data are presented as mean ± SEM. ****$p < 0.0001$, **$p < 0.01$, ns., non-significant. All analytical data are derived from a minimum of three biologically independent experiments, with 'n' indicating the specific number of replicates. Source data are provided as a Source Data file.

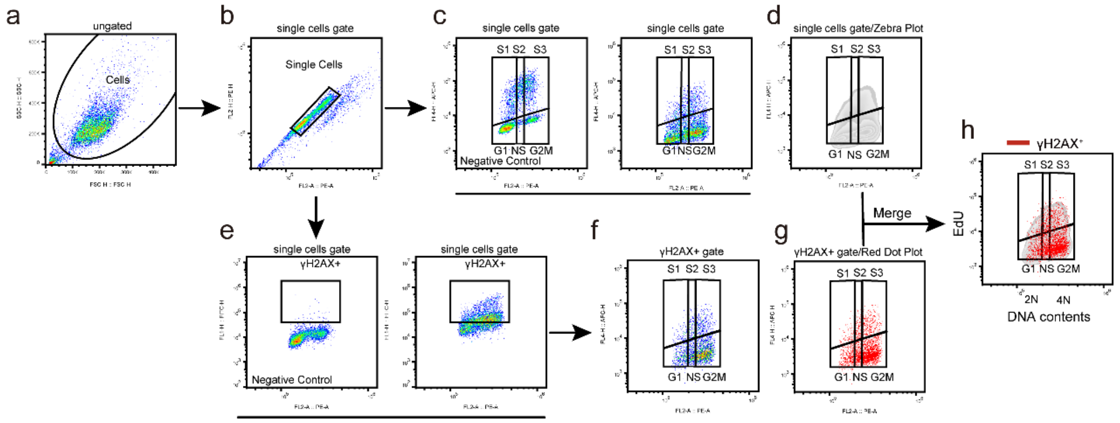


**Fig. S7, Flow-cytometry gating strategy**

Gating hierarchy of a representative sample (OVCAR8 Ctrl and HT 3h). γH2AX, EdU, and PI staining were analyzed by flow cytometry in OVCAR8 cells that were treated with HT for 90 min and allowed to recover at 37 °C for 3h. **a** In FSC-H versus SSC-H, identifcation of the cloud of cell-like events. **b** In PE-A versus PE-H, identifcation of singlets (single cell signals). **c** In PE versus APC, five distinct groups (cells in G1-phase, S1-phase, S2-phase, S31-phase, and G2/M-phases) are shown. **d** The pseudocolored dot plots in c have been changed to grayscale zebra plots. **e** In the FITC (γH2AX-AlexaFluor 488) or PB450 (γH2AX-Pacific Blue) versus PE plot, γH2AX-positive cells is depicted relative to the negative control. **f** In the γH2AX-positive cell population, the APC versus PE axis can be used to visualize the distribution of the five cell cycle phases: S1-phase, S2-phase, S3-phase, G1-phase, Non-replicating S-phase, and G2/M-phase. **g** The pseudocolored dot plots in f have been changed to red dot plots. **h** Merged figure of d and g. The gray zebra pattern represents the overall cell cycle distribution, while the red dot density indicates the percentage of γH2AX-positive cells, with its distribution reflecting the cell cycle distribution of positive cells.

**Supplementary Table 1: Patients clinical information.**

| NO. | Gender | Age Range | Pathology |
|---|---|---|---|
| **1** | | | |
| **2** | | | |
| **3** | Female | 46-65 | HGSOC |
| **4** | | | |
| **5** | | | |

**Supplementary Table 2: Cell lines used in this study.**

| Cell lines | Catalog | Identifier |
|---|---|---|
| Human: HOC7 ovarian cancer cell line | MDACC 's characterized Cell Line Core | N/A |
| Human: OVCAR8 ovarian cancer cell line | MDACC 's characterized Cell Line Core | N/A |
| Human: A2780 ovarian cancer cell line | Procell | HYC3417 |
| Human: ES2 ovarian cancer cell line | ATCC | CRL-1978 |
| Human: SKOV3 ovarian cancer cell line | ATCC | HTB-77 |
| Human: OVCAR3 ovarian cancer cell line | ATCC | HTB-161 |
| Human: Caov3 ovarian cancer cell line | ATCC | HTB-75 |
| Human: OV90 ovarian cancer cell line | ATCC | CRL-11732 |
| Human: TOV-112D ovarian cancer cell line | ATCC | CRL-3593 |
| Human: TOV-21G ovarian cancer cell line | ATCC | CRL-3577 |
| Human: HacaT epidermal keratinocyte | ATCC | PCS-200-011 |
| Human: HUVEC umbilical vein endothelial cells | ATCC | PCS-100-010 |
| Human: IOSE80 ovarian surface epithelium cell line | Cellosaurus | CVCL_5546 |
| Mouse: ID8 ovarian cancer cell line | K. Roby' lab | N/A |
| Mouse: CT26 colon carcinoma cell line | ATCC | CRL-2638 |
| Mouse: MC38 colon adenocarcinoma cell line | MDACC 's characterized Cell Line Core | N/A |

**Supplementary Table 3: The inhibitors used in this study.**

| Inhibitor | Designation | Manufacturers | Catalog | IC50<br>(Cell-free assays) | Secondary targets |
|---|---|---|---|---|---|
| WEE1i | AZD1775 | Selleck | cat#S1525 | 5.2nM | / |
| CDK7i | THZ1 2HCl | Selleck | cat#S7549 | 3.2nM | / |
| GSK-3βi | TWS119 | Selleck | cat#S1590 | 30nM | / |
| CDK4/6i | PD-0332991 | Selleck | cat#S1116 | CDK4/CyclinD1 11nM<br>CDK4/CyclinD3 9nM<br>CDK6/CyclinD2 15nM | / |
| CDK1i | RO-3306 | Selleck | cat#S7747 | 20nM | PKCδ,SGK,ERK |
| Myt1i | PD0166285 | Selleck | cat#S8148 | WEE1 24nM | Myt1, Chk1 |
| p90RSKi | LJI308 | Selleck | cat#S7871 | RSK1 6nM<br>RSK2 5nM<br>RSK3 13nM | / |
| PLK1i | ON-01910 | Selleck | cat#S1362 | 9nM | Bcr-Abl, Flt1,Fyn |
| CHK1/2i | LY2606368 | Selleck | cat#S6385 | 7nM | / |
| Akti | MK-2206 2HC1 | Selleck | cat#S1078 | Akt1 8nM<br>Akt2 12nM<br>Akt3 65nM | / |
| c-Abli | AMN-107 | Selleck | cat#S1033 | ＜30nM | / |
| ATRi | VE-821 | Selleck | cat#S8007 | 13nM | / |
| ATMi | KU55933 | Selleck | cat#S1092 | 12.9nM | / |
| DNA-PKi | KU-57788 | Selleck | cat#S2638 | 14nM | / |
| HIPK2i | Protein kinase inhibitor 1 hydrochloride | MCE | cat#HY-U00439A | HIPK1 136nM<br>HIPK2 74nM | / |

**Supplementary Table 4: The sequences of sgRNAs for CRISPR-Cas9 mediated gene editing.**

| Primer name | Primer sequencs(5'to3') |
|---|---|
| human HSF1-sgRNA-Forward | CACCGCTTGGTGACGCTGTCCTGG |

| | |
|---|---|
| human HSF1-sgRNA-Reverse | AAACCCAGGACAGCGTCACCAAGC |
| human TP53-sgRNA-Forward | CACCGCCCCGGACGATATTGAACAA |
| human TP53-sgRNA-Reverse | AAACTTGTTCAATATCGTCCGGGGC |
| mouse TP53-sgRNA-Forward | CACCGTGGGTAGTTGTAAGCTGACT |
| mouse TP53-sgRNA-Reverse | CACCGTGGGTAGTTGTAAGCTGACT |

**Supplementary Table 5: The sequence information for the plasmid construction.**

| | |
|---|---|
| **pLVX-hPKMYT1-3flag-ZsGreen-Puro plasmid** | Gene Name：human PKMYT1（NM_004203.5）<br>Cloning Vector：pLVX-C-FLAG-mCMV-ZsGreen-IRES-Puro<br>Cloning Strategy：EcoRI+NotI<br>hPKMYT1 sequence：1518bp<br>GAATTCGCCACCATGCTAGAACGGCCTCCTGCACTGGCCATGCCCATGCCCACGGAGGGCACCCCGCCACCTCTG<br>AGTGGCACCCCCATCCCAGTCCCAGCCTACTTCCGCCACGCAGAACCTGGATTCTCCCTCAAGAGGCCCAGGGGG<br>CTCAGCCGGAGCCTCCCACCTCCGCCCCCTGCCAAGGGCAGCATTCCCATCAGCCGCCTCTTCCCTCCTCGGACCC<br>CAGGCTGGCACCAGCTGCAGCCCCGGCGGGTGTCATTCCGGGGCGAGGCCTCAGAGACTCTGCAGAGCCCTGGGT<br>ATGACCCAAGCCGGCCAGAGTCCTTCTTCCAGCAGAGCTTCCAGAGGCTCAGCCGCCTGGGCCATGGCTCCTACG<br>GAGAGGTCTTCAAGGTGCGCTCCAAGGAGGACGGCCGGCTCTATGCGGTAAAGCGTTCCATGTCACCATTCCGGG<br>GCCCCAAGGACCGGGCCCGCAAGTTGGCCGAGGTGGGCAGCCACGAGAAGGTGGGGCAGCACCCATGCTGCGTG<br>CGGCTGGAGCAGGCCTGGGAGGAGGGCGGCATCCTGTACCTGCAGACGGAGCTGTGCGGGCCCAGCCTGCAGCA<br>ACACTGTGAGGCCTGGGGTGCCAGCCTGCCTGAGGCCCAGGTCTGGGGCTACCTGCGGGACACGCTGCTTGCCCT<br>GGCCCATCTGCACAGCCAGGGCCTGGTGCACCTTGATGTCAAGCCTGCCAACATCTTCCTGGGGCCCCGGGGCCG<br>CTGCAAGCTGGGTGACTTCGGACTGCTGGTGGAGCTGGGTACAGCAGGAGCTGGTGAGGTCCAGGAGGGAGACC<br>CCCGCTACATGGCCCCCGAGCTGCTGCAGGGCTCCTATGGGACAGCAGCGGATGTGTTCAGTCTGGGCCTCACCA<br>TCCTGGAAGTGGCATGCAACATGGAGCTGCCCCACGGTGGGGAGGGCTGGCAGCAGCTGCGCCAGGGCTACCTG<br>CCCCCTGAGTTCACTGCCGGTCTGTCTTCCGAGCTGCGTTCTGTCCTTGTCATGATGCTGGAGCCAGACCCCAAGC<br>TGCGGGCCACGGCCGAGGCCCTGCTGGCACTGCCTGTGTTGAGGCAGCCGCGGGCCTGGGGTGTGCTGTGGTGCA<br>TGGCAGCGGAGGCCCTGAGCCGAGGGTGGGCCCTGTGGCAGGCCCTGCTTGCCCTGCTCTGCTGGCTCTGGCATG<br>GGCTGGCTCACCCTGCCAGCTGGCTACAGCCCCTGGGCCCGCCAGCCACCCCGCCTGGCTCACCACCCTGCAGTTT<br>GCTCCTGGACAGCAGCCTCTCCAGCAACTGGGATGACGACAGCCTAGGGCCTTCACTCTCCCCTGAGGCTGTCCTG<br>GCCCGGACTGTGGGGAGCACCTCCACCCCCCGGAGCAGGTGCACACCCAGGGATGCCCTGGACCTAAGTGACATC<br>AACTCAGAGCCTCCTCGGGGCTCCTTCCCCTCCTTTGAGCCTCGGAACCTCCTCAGCCTGTTTGAGGACACCCTAG<br>ACCCAACCAGCGGCCGC |

| | |
|---|---|
| **pLVX-mPkmyt1-3flag-ZsGreen-Puro plasmid** | Gene Name: mouse Pkmyt1（NM_023058.3）<br>Cloning Vector: pLVX-C-FLAG-mCMV-ZsGreen-IRES-Puro<br>Cloning Strategy: EcoRI+NotI<br>mPkmyt1 synthesis sequence: 1491bp<br>GAATTCGCCACCATGACCATGCCCACCGAGGGCACCCCACCACCCCTAAGTGGTACCCCCATCCCAGTTCCAGCTT<br>ACTTCCGACACGCAGAGCCTGGTTTCTCCCTCAAAAGGCCTGGGGGCCTCAGTCGGAGCCTCCCACCTCGGCCCCC<br>TGCCAAGGGCTGCATCCCTGTCAGCCGTCTATTCCCCCCTCGCACCCCAGGCTGGCACCAGCCCCAGCCCCGGAG<br>GGTGTCTTTCCTGTGTGAGACCTCAGAGCCCCTGCAGAGTCCTGGGTATGACCCGAGCCGGCCCGAGTCCTTCTTT<br>CAGCAGAACTTCCAGAGGCTCAGCCGCCTGGGTCATGGCTCATATGGAGAAGTCTTCAAGGTGCGCTCTAAGGAA<br>GATGGGCGACTCTATGCTGTTAAGCGCTACATGTCGCCATTCCGCGGCCCCAAAGACCGAACTCGTAAACTGGCT<br>GAGGTAGGTGGCCATGAGAAAGTGGGGCAGCATCCACACTGCGTGAGACTGGAGCGGGCCTGGGAGGAGGGTGG<br>CATCCTATACCTGCAGACAGAACTCTGCGGGCCCAGCCTGCAGCAACACTGTGAAGCCTGGGGGGCCAGCCTGCC<br>AGAGGCCCAGGTCTGGGGCTACTTGCGGGACATTCTTCTGGCTCTGGACCATCTACATAGTCAAGGCCTAGTTCAC<br>CTTGATGTCAAGCCTGCCAACATCTTCTTGGGCCCCCGGGGCCGCTGCAAGCTGGGCGACTTTGGACTACTGGTGG<br>AGCTGGGTTCAGCCGGTGCTGGCGAGGCCCAGGAGGGAGATCCTCGCTACATGGCCCCAGAACTGCTGCAGGGCT<br>CTTATGGGACAGCAGCAGATGTGTTCAGTCTGGGTCTCACCATCTTGGAAGTGGCCTGTAACATGGAACTGCCCC<br>ATGGTGGGGAGGGCTGGCAGCAGCTGCGCCAGGGATACTTGCCCCCTGAGTTCACTGCTGGTCTGTCTTCTGAGCT<br>GCGTTCTGTCCTCGCCATGATGCTGGAGCCTGACCCCCAGCTTCGAGCCACAGCTGAGGCCCTGTTGGCCTTACCC<br>ATGCTGAGGCAGCCACGTCCCTGGAATGTTCTGTGGTATATGGCTGCGGAAGCCCTAAGTCGAGGCTGGGCCCTG<br>TGGCAGGCCCTGGTCACTCTGCTCTGCTGGCTCTGGCACGGGCTGGTGCATCCTGCCAGTTGGCTGCAGCCTCCAG<br>GCCCACCGGCCACACCACCTGGCTCTCCACCTTGCAGCCCCCTCCTGGACAGCACCCTCTCCAGCAGCTGGGATAA<br>TGACAGCATAGGTCCCTCACTCTCCCCAGAGACCGTCCTGTCCCGGATCACTAGAAGAACCTCTACCCCTCGGGGC<br>AGGTACATACCTAGGGATGCCCTGGACCTAACTGATGTGGACTCAGAGCCTCCAAGAGGTCCCTGCCCCACCTTT<br>GAGCCAAGGAACCTCCTCAGCCTGTTTGAGGACTCCCTAGACCCAGCCGGCGGCCGC |

**Supplementary Table 6: The sequences of siRNAs used in this study.**

| siRNA | Target sequencs |
|---|---|
| genOFFTM st-h-WEE1_001 | GTGTCGTCGTAGAAAGAGA |
| genOFFTM st-h-WEE1_002 | GTCCCGGTATACAACAGAA |
| genOFFTM st-h-WEE1_003 | GTCCCGGTATACAACAGAA |
| si-m-Myt1_001 | GCTTGAGGTTCCACCTTAT |
| si-m-Myt1_002 | GAGCTGCTACAGCAAAGAT |
| si-m-Myt1_002 | GAGCTGCTACAGCAAAGAT |

**Supplementary Table 7: The antibodies used in this study.**

| Antibody | Source | Identifier | Application | Dilution Ratio |
|---|---|---|---|---|
| Rabbit anti-γH2AX（Ser139） | ABclonal | Cat#AP0687 RRID:AB_2863808 | IF | 1:200 |
| | | | WB | 1:1000 |
| | | | IHC | 1:200 |
| Alexa Fluor 488 anti-rabbit | Jackson Immunoresearch | Cat# 711-545-152 RRID: AB_2313584 | IF | 1:200 |
| Rat anti-BrdU（clone BU1/75（ICR1）） | Abcam | Cat# ab6326 RRID:AB_2313786 | IF | 1:200 |
| | | | IF (DNA fiber) | 1:300 |
| Mouse anti-BrdU（clone B44） | BD | Cat#347580 RRID:AB_10015219 | IF (DNA fiber) | 1:50 |
| AlexaFluor 488 anti-Mouse | Jackson Immunoresearch | Cat# 715-545-151 RRID:AB_2341099 | Flow Cyt | 1:200 |
| AlexaFluor 488 anti-Rat | Jackson Immunoresearch | Cat# 712-545-153 RRID: AB_2340684 | IF (DNA fiber) | 1:150 |
| AlexaFluor 594 anti-Mouse | Jackson Immunoresearch | Cat# 715-585-151 RRID: AB_2340855 | IF (DNA fiber) | 1:150 |
| | | | IF | 1:200 |
| Rabbit anti- phospho-Histone H3（Ser10） | Abcam | Cat# ab5176 RRID:AB_304763 | IF | 1:200 |
| Mouse anti-γH2AX（Ser139） | CST | Cat#80312 RRID:AB_2799949 | Flow Cyt | 1:200 |
| | | | IF | 1:200 |
| AlexaFluor 594 anti-Rat | Jackson Immunoresearch | Cat# 712-585-153 RRID: AB_2340689 | IF | 1:200 |
| Rabbit anti- PKMYT1 | ABclonal | Cat# A20525 RRID:AB_3065595 | WB | 1:1000 |
| Rabbit anti- phospho-CDK1（Tyr15） | ABclonal | Cat# AP0016 RRID:AB_2770978 | IHC | 1:200 |
| | | | WB | 1:1000 |
| Rabbit anti- phospho-CDK1（Thr14） | ABclonal | Cat# AP0015 RRID:AB_2770976 | WB | 1:1000 |
| Rabbit anti- phospho-PPP1Ca（Thr320） | Abcam | Cat# ab62334 RRID:AB_956236 | WB | 1:1000 |
| Rabbit anti-CDK1 | ABclonal | Cat# A0220 RRID:AB_2757034 | WB | 1:1000 |
| Rabbit anti-RRM2 | ABclonal | Cat# A3424 RRID:AB_2863055 | WB | 1:1000 |
| Rabbit anti-cleaved-Caspase-3 | CST | Cat#9661 RRID:AB_2341188 | WB | 1:1000 |

| Antibody | Source | Identifier | Application | Dilution Ratio |
| --- | --- | --- | --- | --- |
| Rabbit anti-cleaved-PARP（Asp214） | CST | Cat#9541S RRID:AB_331426 | WB | 1:1000 |
| Rabbit anti-β-Tubulin | ABclonal | Cat# A12289 RRID:AB_2861647 | WB | 1:1000 |
| Rabbit anti-GAPDH | ABclonal | Cat# A19056 RRID:AB_2862549 | WB | 1:1000 |
| Rabbit anti-β-Actin | ABclonal | Cat# AC006 RRID:AB_2768236 | WB | 1:1000 |
| HRP Goat Anti-Rabbit（H+L） | ABclonal | Cat# AS014 RRID:AB_2769854 | WB | 1:5000 |
| Mouse Anti-Ki67 | CST | Cat# 9449 RRID:AB_2687446 | IHC | 1:100 |
| Rabbit anti-PKMYT1 | Abcam | Cat#ab307146 | WB | 1:1000 |
| Rabbit anti-PKMYT1 | Abcam | Cat#ab200387 | IHC | 1:200 |
| Rabbit anti-WEE1 | abclonal | Cat# A16256 RRID:AB_2772894 | WB | 1:1000 |
| Rabbit anti-P53 | Proteintech | Cat#10442-1-AP RRID:AB_2206609 | WB | 1:1000 |
| Rabbit anti-HSF1 | CST | Cat #12972 RRID:AB_2798072 | WB | 1:1000 |
| Pacific Blue anti-Mouse | Thermo Fisher | Cat # P31582 RRID:AB_10374586 | Flow Cyt | 1:200 |
