# Supplementary material for: Comprehensive multi-omics analysis reveals WEE1 as a synergistic lethal target with hyperthermia through CDK1 super-activation
Source: Nat Commun. 2024 Mar 7;15:2089. doi: 10.1038/s41467-024-46358-w (PMC10920785; doi:10.1038/s41467-024-46358-w)
Supplement: Supplementary file 3 — Description of Additional Supplementary Files [file 41467_2024_46358_MOESM3_ESM.pdf]

Description of Additional Supplementary Files

File Name: Supplementary Data 1
Description: Differential expression analysis of transcriptomic data

File Name: Supplementary Data 2
Description: Differential analysis of quantitative proteomics data

File Name: Supplementary Data 3
Description: Differential analysis of phosphoproteomic data
