# Supplementary material for: Comprehensive multi-omics analysis reveals WEE1 as a synergistic lethal target with hyperthermia through CDK1 super-activation
Source: Nat Commun. 2024 Mar 7;15:2089. doi: 10.1038/s41467-024-46358-w (PMC10920785; doi:10.1038/s41467-024-46358-w)
Supplement: Supplementary file 7 — Reporting Summary [file 41467_2024_46358_MOESM7_ESM.pdf]

nature portfolio

Corresponding author(s): Chaoyang Sun, Gang Chen, Jing Li

Last updated by author(s): Jan 17, 2024

Reporting Summary

Nature Portfolio wishes to improve the reproducibility of the work that we publish. This form provides structure for consistency and transparency in reporting. For further information on Nature Portfolio policies, see our Editorial Policies and the Editorial Policy Checklist.

Statistics

For all statistical analyses, confirm that the following items are present in the figure legend, table legend, main text, or Methods section.

n/a	Confirmed	
☐	☒	The exact sample size (*n*) for each experimental group/condition, given as a discrete number and unit of measurement
☐	☒	A statement on whether measurements were taken from distinct samples or whether the same sample was measured repeatedly
☐	☒	The statistical test(s) used AND whether they are one- or two-sided<br>*Only common tests should be described solely by name; describe more complex techniques in the Methods section.*
☒	☐	A description of all covariates tested
☐	☒	A description of any assumptions or corrections, such as tests of normality and adjustment for multiple comparisons
☐	☒	A full description of the statistical parameters including central tendency (e.g. means) or other basic estimates (e.g. regression coefficient) AND variation (e.g. standard deviation) or associated estimates of uncertainty (e.g. confidence intervals)
☐	☒	For null hypothesis testing, the test statistic (e.g. *F*, *t*, *r*) with confidence intervals, effect sizes, degrees of freedom and *P* value noted<br>*Give P values as exact values whenever suitable.*
☒	☐	For Bayesian analysis, information on the choice of priors and Markov chain Monte Carlo settings
☒	☐	For hierarchical and complex designs, identification of the appropriate level for tests and full reporting of outcomes
☐	☒	Estimates of effect sizes (e.g. Cohen's *d*, Pearson's *r*), indicating how they were calculated

Our web collection on statistics for biologists contains articles on many of the points above.

Software and code

Policy information about availability of computer code

Data collection	RNA-seq data was generated by sequencing performed on the IDNBSEQ-G400 platform.<br>The proteomics and phosphoproteomics samples were fractionated into various fractions using high-pH reverse-phase high-performance liquid chromatography (HPLC) with an Agilent 300 Extend C18 column (5 μm particles, 4.6 mm ID, 250 mm length).<br>OD values were assessed at a wavelength of 450 nm utilizing the SpectraMax ABS Plus Microplate Reader.<br>The flow cytometry samples were analyzed on a Beckman Coulter CytoFLEX Flow Cytometer, and flow cytometry data were collected using CytExpert (version 2.4).<br>In the DNA fiber assay, slides were mounted using the Operetta CLS High-Content Analysis System (PerkinElmer), and image data were collected with Harmony (version 2.9) software.<br>In immunofluorescence staining, cells were observed and imaged with the ImageXpress Micro Confocal High-Content Analysis System (Molecular Devices).<br>In Western blotting, specific bands were visualized using the ChemiDoc Imaging System (Bio-Rad).<br>In IHC staining, slides were recognized and images were captured using the Slide Scan System SQS-600P 40X/80X (Teksqray).<br>In vivo imaging was conducted in PerkinElmer IVIS Lumina III System.
Data analysis	For RNA-seq raw data, the adaptor was trimmed with Trim Galore (v.0.6.7) and then aligned to the human genome (hg38) with STAR (v.2.7.10a) The read count was generated by subread (v.2.0.1) and the transcripts per million (TPMs) of genes were quantified using RSEM (v.1.3.1). Differential gene expression analysis was performed in R using DESeq2. The R package clusterProfiler was used for pathway enrichment. The resulting MS/MS data were processed using the MaxQuant search engine (v.1.6.15.0) against the Homo_sapiens_9606_SP_20201214.fasta (20395 entries). Kinase activity scores were inferred from phosphorylation sites by employing PTM signature enrichment analysis (PTM-SEA) using the PTM signatures database (PTMsigDB, v1.9.0). The protein–protein interaction (PPI)

network of the candidate genes was constructed using the Search Tool for the Retrieval of Interacting Genes (STRING) database (https://string-db.org/) and visualized using Cytoscape (v.3.1.2).
In the clonogenic assay, colony numbers were quantified using Image J (version 1.53k).
FlowJo V10 software was utilized for the analysis and visualization of the flow cytometry data.
In immunofluorescence staining, quantification of cell nuclei and positive cells was performed using ImageJ software (version 1.53k).
In Western blotting, specific bands processed with Image Lab software (version 6.0.1).
Statistical analysis and data plotting were conducted using GraphPad Prism (version 9.0.0) software

For manuscripts utilizing custom algorithms or software that are central to the research but not yet described in published literature, software must be made available to editors and reviewers. We strongly encourage code deposition in a community repository (e.g. GitHub). See the Nature Portfolio guidelines for submitting code & software for further information.

Data

Policy information about availability of data

All manuscripts must include a data availability statement. This statement should provide the following information, where applicable:

- Accession codes, unique identifiers, or web links for publicly available datasets
- A description of any restrictions on data availability
- For clinical datasets or third party data, please ensure that the statement adheres to our policy

PTM signatures database (PTMsigDB, v1.9.0) was downloaded from hhttps://proteomics.broadapps.org/ptmsigdb/.
All mass spectrometry proteomics and phosphoproteomics data have been deposited to the ProteomeXchange Consortium via the PRIDE partner repository with the dataset identifier PXD040849 [https://www.ebi.ac.uk/pride/archive/projects/PXD040849].
RNA-seq data are available in the Gene Expression Omnibus (GEO) database under accession code GSE227393 [https://www.ncbi.nlm.nih.gov/geo/query/acc.cgi?acc=GSE227393].
Differential expression analysis of transcriptomic data is elaborated in Supplementary Data 1 for further details.
Differential analysis of quantitative proteomics data is elaborated in Supplementary Data 2 for further details.
Differential analysis of phosphoproteomic data is elaborated in Supplementary Data 3 for further details.
The data are publicly available as of the date of publication.

Research involving human participants, their data, or biological material

Policy information about studies with human participants or human data. See also policy information about sex, gender (identity/presentation), and sexual orientation and race, ethnicity and racism.

Reporting on sex and gender	Our research focuses on the treatment of tumors occurring in the ovaries and we have not taken sex and gender into account in the design of the study. The five ovarian cancer samples we collected are from five ovarian cancer patients who underwent cytoreductive surgery or exploratory surgery at Tongji Hospital, Tongji Medical College, Huzhong University of Science and Technology.
Reporting on race, ethnicity, or other socially relevant groupings	Our study did not involve considerations on race ethnicity or other socially relevant groups. Race and ethnicity data were not collected.
Population characteristics	All patients were initially diagnosed with ovarian cancer or highly suspected ovarian carcinoma upon admission. Following admission, patients underwent examinations and treatments in accordance with the clinical pathway for ovarian cancer. Upon discharge, all patients received a confirmed diagnosis of High-Grade Serous Ovarian Cancer. The five female ovarian cancer patients in this study had the following ages: Patient 1: 57 years old, Patient 2: 46 years old, Patient 3: 65 years old, Patient 4: 62 years old, and Patient 5: 52 years old. Additionally, other clinical details, such as Grade, Figo stage, specific pathology, surgeries, postoperative adjuvant treatments, and CA125 level changes, are outlined in Supplementary Table 1.
Recruitment	The ovarian tumor samples in this study were ethically sourced from individuals undergoing cytoreductive or exploratory surgery at Tongji Hospital, Tongji Medical College, Huazhong University of Science and Technology. Stringent adherence to ethical regulations governing human participant research was maintained, with all tissue acquisition procedures and experimental protocols receiving explicit approval from the Institutional Review Board (IRB) of Tongji Hospital (Permit Number: S080). The recruitment process involved informing potential participants about the study and obtaining their informed consent. We acknowledge that the complete randomization of participant selection and the relatively small sample size may introduce biases into our study. It is imperative, however, to clarify that these five human-sourced HGSOC samples serve a supplementary role in validating our research rather than constituting its primary focus. Emphasis is placed on the supplementary role of the five human-sourced HGSOC samples, with a commitment to transparently addressing potential biases and encouraging cautious interpretation and generalization beyond the recruited population.
Ethics oversight	The study's tissue acquisition procedures and experimental protocols were approved by the Institutional Review Board (IRB) of Tongji Hospital (Permit Number: S080), with informed consent from all participants.

Note that full information on the approval of the study protocol must also be provided in the manuscript.

Field-specific reporting

Please select the one below that is the best fit for your research. If you are not sure, read the appropriate sections before making your selection.

☒ Life sciences ☐ Behavioural & social sciences ☐ Ecological, evolutionary & environmental sciences

For a reference copy of the document with all sections, see nature.com/documents/nr-reporting-summary-flat.pdf

Life sciences study design

All studies must disclose on these points even when the disclosure is negative.

Sample size	No statistical methods were used to pre-determine sample size. The sample size for each experiment is provided in the figures or legends in the main manuscript and supplementary file. For sequencing, samples were prepared in three biological replicates. For experiments related to primary cell culture under in vitro conditions, the relevant experiments for the five samples were conducted independently. For in vivo mouse models, each group consisted of at least 3 mice. For experiments with live cells, each group was repeated at least three times. These sizes have previously been shown to be sufficiently powered to determine statistical differences in the mean values of our investigated parameters.
Data exclusions	No data were excluded from analysis, except for necessary data quality control steps during the initial RNA-seq, proteomics, and phosphoproteomics data processing.
Replication	The replication number is indicated in the legend of corresponding figures where applicable.
Randomization	Cells and mice were randomly allocated into experimental groups.
Blinding	All the control and experimental group of mice/cells were grown under identical conditions.The investigators were not blinded as propercontrols were already included during experiment design.

Reporting for specific materials, systems and methods

We require information from authors about some types of materials, experimental systems and methods used in many studies. Here, indicate whether each material, system or method listed is relevant to your study. If you are not sure if a list item applies to your research, read the appropriate section before selecting a response.

Materials & experimental systems

n/a	Involved in the study	
☐	☒	Antibodies
☐	☒	Eukaryotic cell lines
☒	☐	Palaeontology and archaeology
☐	☒	Animals and other organisms
☒	☐	Clinical data
☒	☐	Dual use research of concern
☒	☐	Plants

Methods

n/a	Involved in the study	
☒	☐	ChIP-seq
☐	☒	Flow cytometry
☒	☐	MRI-based neuroimaging

Antibodies

Antibodies used

Rabbit anti-γH2AX?Ser139? ABclonal Cat#AP0687, RRID:AB_2863808
Alexa Fluor 488 anti-rabbit Jackson Immunoresearch Cat# 711-545-152, RRID: AB_2313584
Rat anti-BrdU clone BU1/75 ICR1 Abcam Cat# ab6326, RRID:AB_2313786
Mouse anti-BrdU clone B44 BD Cat#347580, RRID:AB_10015219
AlexaFluor 488 anti-Mouse Jackson Immunoresearch Cat# 715-545-151, RRID:AB_2341099
AlexaFluor 488 anti-Rat Jackson Immunoresearch Cat# 712-545-153, RRID: AB_2340684
AlexaFluor 594 anti-Mouse Jackson Immunoresearch Cat# 715-585-151, RRID: AB_2340855
Rabbit anti- phospho-Histone H3 Ser10 Abcam Cat# ab5176, RRID:AB_304763
Mouse anti-γH2AX?Ser139? CST Cat#80312, RRID:AB_2799949
AlexaFluor 594 anti-Rat Jackson Immunoresearch Cat# 712-585-153, RRID: AB_2340689
Rabbit anti- PKMYT1 ABclonal Cat# A20525, RRID:AB_3065595
Rabbit anti- phospho-CDK1 Tyr15 ABclonal Cat# AP0016, RRID:AB_2770978
Rabbit anti- phospho-CDK1 Thr14 ABclonal Cat# AP0015, RRID:AB_2770976
Rabbit anti- phospho-PPP1Ca Thr320 Abcam Cat# ab62334, RRID:AB_956236
Rabbit anti-CDK1 ABclonal Cat# A0220, RRID:AB_2757034
Rabbit anti-RRM2 ABclonal Cat# A3424, RRID:AB_2863055
Rabbit anti-cleaved-Caspase-3 CST Cat#9661, RRID:AB_2341188
Rabbit anti-cleaved-PARP Asp214 CST Cat#9541S, RRID:AB_331426

Rabbit anti-β-Tubulin ABclonal Cat# A12289, RRID:AB_2861647
Rabbit anti-GAPDH ABclonal Cat# A19056, RRID:AB_2862549
Rabbit anti-β-Actin ABclonal Cat# AC006, RRID:AB_2768236
HRP Goat Anti-Rabbit H+L ABclonal Cat# AS014, RRID:AB_2769854
Rabbit Anti-Ki67 CST Cat#9129, RRID:AB_2687446
Rabbit anti-PKMYT1 Abcam Cat#ab307146
Rabbit anti-PKMYT1 Abcam Cat#ab200387
Rabbit anti-WEE1 abclonal Cat# A16256, RRID:AB_2772894
Rabbit anti-P53 Proteintech Cat#10442-1-AP, RRID:AB_2206609
Rabbit anti-HSF1 CST Cat #12972, RRID:AB_2798072
Pacific Blue anti-Mouse Thermo Fisher Cat # P31582, RRID:AB_10374586

Validation

All the antibodies tested in the manuscript has been published or described previously. Validation and citation information can be found on the manufacturer's website. Their original links are listed in the below:
Rabbit anti-γH2AX?Ser139? ABclonal Cat#AP0687, IF/WB/IHC, https://abclonal.com.cn/catalog/AP0687
Alexa Fluor 488 anti-rabbit Jackson Immunoresearch Cat# 711-545-152, IF, https://www.jacksonimmuno.com/catalog/products/711-545-152
Rat anti-BrdU clone BU1/75 ICR1 Abcam Cat# ab6326, IF, https://www.abcam.cn/products%2fprimary-antibodies%2fbrdu-antibody-bu175-icr1-proliferation-marker-ab6326.html
Mouse anti-BrdU clone B44 BD Cat#347580, IF, https://www.bdbiosciences.com/en-us/products/reagents/flow-cytometry-reagents/clinical-discovery-research/single-color-antibodies-ruo-gmp/purified-mouse-anti-brdu.347580
AlexaFluor 488 anti-Mouse Jackson Immunoresearch Cat# 715-545-151, Flow Cyt, https://www.jacksonimmuno.com/catalog/products/715-545-151
AlexaFluor 488 anti-Rat Jackson Immunoresearch Cat# 712-545-153, IF, https://www.jacksonimmuno.com/catalog/products/712-545-153
AlexaFluor 594 anti-Mouse Jackson Immunoresearch Cat# 715-585-151, IF, https://www.jacksonimmuno.com/catalog/products/715-585-151
Rabbit anti- phospho-Histone H3 Ser10 Abcam Cat# ab5176, IF, https://www.abcam.cn/products/primary-antibodies/histone-h3-phospho-s10-antibody-ab5176.html
Mouse anti-γH2AX?Ser139? CST Cat#80312, IF/Flow Cyt, https://www.cellsignal.com/products/primary-antibodies/phospho-histone-h2a-x-ser139-d7t2v-mouse-mab/80312
AlexaFluor 594 anti-Rat Jackson Immunoresearch Cat# 712-585-153, IF, https://www.jacksonimmuno.com/catalog/products/712-585-153
Rabbit anti- PKMYT1 ABclonal Cat# A20525, WB, https://abclonal.com.cn/catalog/A20525
Rabbit anti- phospho-CDK1 Tyr15 ABclonal Cat# AP0016, WB/IHC, https://abclonal.com.cn/catalog/AP0016
Rabbit anti- phospho-CDK1 Thr14 ABclonal Cat# AP0015, WB, https://abclonal.com.cn/catalog/AP0015
Rabbit anti- phospho-PPP1Ca Thr320 Abcam Cat# ab62334, WB, https://www.abcam.cn/products/primary-antibodies/ppp1appp1ca-phospho-t320-antibody-ep1512y-ab62334.html
Rabbit anti-CDK1 ABclonal Cat# A0220, WB, https://abclonal.com.cn/catalog/A0220
Rabbit anti-RRM2 ABclonal Cat# A3424, WB, https://abclonal.com.cn/catalog/A3424
Rabbit anti-cleaved-Caspase-3 CST Cat#9661, WB, https://www.cellsignal.cn/products/primary-antibodies/cleaved-caspase-3-asp175antibody/9661
Rabbit anti-cleaved-PARP Asp214 CST Cat#9541S, WB, https://www.cellsignal.com/products/primary-antibodies/cleaved-parp-asp214-antibody-human-specific/9541
Rabbit anti-β-Tubulin ABclonal Cat# A12289, WB, https://abclonal.com.cn/catalog/A12289
Rabbit anti-GAPDH ABclonal Cat# A19056, WB, https://abclonal.com.cn/catalog/A19056
Rabbit anti-β-Actin ABclonal Cat# AC006, WB, https://abclonal.com.cn/catalog/AC006
HRP Goat Anti-Rabbit H+L ABclonal Cat# AS014, WB, https://abclonal.com.cn/catalog/AS014
Rabbit Anti-Ki67 CST Cat#9449, IHC, https://www.cellsignal.cn/products/primary-antibodies/ki-67-8d5-mouse-mab/9449
Rabbit anti-PKMYT1 Abcam Cat#ab307146, WB, https://www.abcam.cn/products/primary-antibodies/pkmyt1-antibody-epr27155-51-ab307146.html
Rabbit anti-PKMYT1 Abcam Cat#ab200387, IHC, https://www.abcam.cn/products/primary-antibodies/pkmyt1-antibody-ab200387.html
Rabbit anti-WEE1 abclonal Cat# A16256, WB, https://abclonal.com.cn/catalog/A16256
Rabbit anti-P53 Proteintech Cat#10442-1-AP, WB, https://www.ptgcn.com/Products/P53-Antibody-10442-1-AP.htm
Rabbit anti-HSF1 CST Cat #12972, WB, https://www.cellsignal.cn/products/primary-antibodies/hsf1-d3l8i-rabbit-mab/12972
Pacific Blue anti-Mouse Thermo Fisher Cat # P31582, Flow Cyt, https://www.thermofisher.cn/cn/zh/antibody/product/Goat-anti-Mouse-IgG-H-L-Cross-Adsorbed-Secondary-Antibody-Polyclonal/P31582

Eukaryotic cell lines

Policy information about cell lines and Sex and Gender in Research

Cell line source(s)

Human: HOC7 ovarian cancer cell line MDACC 's characterized Cell Line Core
Human: OVCAR8 ovarian cancer cell line MDACC 's characterized Cell Line Core
Human: A2780 ovarian cancer cell line Procell HYC3417
Human: ES2 ovarian cancer cell line ATCC CRL-1978
Human: SKOV3 ovarian cancer cell line ATCC HTB-77
Human: OVCAR3 ovarian cancer cell line ATCC HTB-161
Human: Caov3 ovarian cancer cell line ATCC HTB-75
Human: OV90 ovarian cancer cell line ATCC CRL-11732
Human: TOV-112D ovarian cancer cell line ATCC CRL-3593

	Human: TOV-21G ovarian cancer cell line ATCC CRL-3577<br>Human: HacaT epidermal keratinocyte ATCC PCS-200-011<br>Human: HUVEC umbilical vein endothelial cells ATCC PCS-100-010<br>Human: IOSE80 ovarian surface epithelium cell line Cellosaurus CVCL_5546<br>Mouse: ID8 ovarian cancer cell line K. Roby' lab N/A<br>Mouse: CT26 colon carcinoma cell line ATCC CRL-2638<br>Mouse: MC38 colon adenocarcinoma cell line MDACC 's characterized Cell Line Core
Authentication	Cell lines were validated by STR profiling.
Mycoplasma contamination	All cell lines tested negative for mycoplasma contamination.
Commonly misidentified lines<br>(See ICLAC register)	None

Animals and other research organisms

Policy information about studies involving animals; ARRIVE guidelines recommended for reporting animal research, and Sex and Gender in Research

Laboratory animals	Female C57BL/6 and NOD mice (6-7 weeks old) were procured from Gempharmatech Co., Ltd. The animals were maintained in sterile conditions at a temperature of 20–25°C with 50% humidity and a 12-hour light-dark cycle. Cages, covers, bedding, food, and water were changed and sterilized on a weekly basis.
Wild animals	None
Reporting on sex	Sex was not considered in these animal experiments. Our ovarian cancer peritoneal dissemination model was constructed using female mice with intact ovaries.
Field-collected samples	None
Ethics oversight	All related mouse study protocols received approval from the Institutional Animal Care and Use Committee (IACUC) of Tongji Hospital (Permit Number: TJH-202203009) and were conducted following the Chinese Council on Animal Care's guidelines for animal care and use.

Note that full information on the approval of the study protocol must also be provided in the manuscript.

Plants

Seed stocks	*Report on the source of all seed stocks or other plant material used. If applicable, state the seed stock centre and catalogue number. If plant specimens were collected from the field, describe the collection location, date and sampling procedures.*
Novel plant genotypes	*Describe the methods by which all novel plant genotypes were produced. This includes those generated by transgenic approaches, gene editing, chemical/radiation-based mutagenesis and hybridization. For transgenic lines, describe the transformation method, the number of independent lines analyzed and the generation upon which experiments were performed. For gene-edited lines, describe the editor used, the endogenous sequence targeted for editing, the targeting guide RNA sequence (if applicable) and how the editor was applied.*
Authentication	*Describe any authentication procedures for each seed stock used or novel genotype generated. Describe any experiments used to assess the effect of a mutation and, where applicable, how potential secondary effects (e.g. second site T-DNA insertions, mosiacism, off-target gene editing) were examined.*

Flow Cytometry

Plots

Confirm that:

☒ The axis labels state the marker and fluorochrome used (e.g. CD4-FITC).

☒ The axis scales are clearly visible. Include numbers along axes only for bottom left plot of group (a 'group' is an analysis of identical markers).

☒ All plots are contour plots with outliers or pseudocolor plots.

☒ A numerical value for number of cells or percentage (with statistics) is provided.

Methodology

Sample preparation	In flow cytometry for apoptosis, cells were harvested and then stained with Annexin V and propidium iodide (PI) using the FITC Annexin V Apoptosis Detection Kit I (BD Biosciences, 556547). In flow cytometry for cell cycle anlysis with EdU incorporation and γH2AX detection, the EdU-647 Cell Proliferation Assay Kit (Beyotime, C0081S) were used according to the manufacturer's instructions. Briefly, cells were incubated in medium with a final concentration of 10 μM EdU for 2 h, then

harvested for fixation in 4% polyformaldehyde for 15 min. After washing with PBS, cells were permeabilized with 0.3% Triton X-100 for 10 min and blocked using 3% BSA in PBS. The cells were then labeled with Azide 647 click addition solution at RT for 30 min in the dark, and subsequently rinsed with PBS. Cells were then incubated with specific antibodies against γH2AX (CST, 80312, 1:200) for 90 min at RT. Next, cells were washed with PBS and incubated with a secondary antibody conjugated to AlexaFluor 488 (Jackson Immunoresearch, 715-585-151, 1:200) or Pacific Blue (Thermo Fisher, P31582, 1:200) for 1 h. Finally, cells were stained with PI which contained 100 mg/mL RNase A (BD Biosciences, 550825).

Instrument

Beckman Coulter CytoFLEX Flow Cytometer

Software

CytExpert (version 2.4)

Cell population abundance

Sample was collected with a minimum of 10,000 cells.

Gating strategy

In flow cytometry for apoptosis, the FACS sequential gating strategies were as follows: The starting cell population was selected through FSC/SSC gates, where debris and dead cells with lower forward scatter were excluded. The density plot was segmented into four quadrants to identify cells that were either single positive for Annexin V-FITC, double negative, or double positive for Annexin V-FITC and PI. The relative proportions of early and late apoptosis cells were quantified by placing gates around these distinct populations. Apoptosis levels were quantified by calculating the fractional difference in Annexin V-FITC positive (early) and Annexin V-FITC/PI double-positive (advanced) populations between treated and untreated samples. At least 1×104 cells were collected to determine the percentage of apoptotic cells.
In flow cytometry for cell cycle anlysis with EdU incorporation and γH2AX detection, the initial cell population was chosen based on FSC/SSC gates, excluding debris and dead cells with lower forward scatter. Gating on single cells and excluding clumps or doublets on a plot of PE-A (forward scatter area) vs PE-H (forward scatter height). In the APC (EdU-Azide 647) versus PE (DNA contents-PI) plot, five distinct groups are displayed, representing cells in S1-phase, S2-phase, S3-phase, G1-phase, Non-replicating S-phase, and G2/M-phase. In the FITC (γH2AX-AlexaFluor 488) or PB450 (γH2AX- Pacific Blue) versus PE plot, γH2AX-positive cells are depicted relative to the negative control. Within the γH2AX-positive cell population, the APC versus PE axis can be used to visualize the distribution of the five cell cycle phases: S1-phase, S2-phase, S3-phase, G1-phase, Non-replicating S-phase, and G2/M-phase.

☒ Tick this box to confirm that a figure exemplifying the gating strategy is provided in the Supplementary Information.
